# Supplementary material for: Effectiveness of intravenous lidocaine in preventing postoperative nausea and vomiting in pediatric patients: A systematic review and meta-analysis
Source: PLoS One. 2020 Jan 28;15(1):e0227904. doi: 10.1371/journal.pone.0227904 (PMC6986726; doi:10.1371/journal.pone.0227904)
Supplement: S1 Text — (DOCX) [file pone.0227904.s003.docx]

S1 text. Search strategy for PubMed.

The following PubMed search strategy was established:

(“lidocaine” [Mh] OR “lidocaine” [All] OR “lignocaine” [All] OR “xylocaine” [All]) AND (“child”[Mh] OR “child”[All] OR “children”[All] OR “pediatrics”[Mh] OR “pediatrics”[All] OR “pediatric”[All] OR “paediatrics”[All] OR “paediatric”[All] OR “infant”[Mh] OR “infant”[All] OR “adolescent”[Mh] OR “adolescent”[All]) AND (“anesthesia” [Mh] OR “anesthesia” [All] OR “anaesthesia” [All]) AND (“perioperative period"[Mh] OR "perioperative care"[Mh] OR “perioperative” [All] OR “intraoperative” [All] OR “preoperative” [All] OR “postoperative” [All]) AND (randomized controlled trial [pt] OR controlled clinical trial [pt] OR randomized [tiab] OR placebo [tiab] OR drug therapy [sh] OR randomly [tiab] OR trial [tiab] OR groups [tiab]) NOT (animals [mh] NOT humans [mh])
